# Inhibition of EphA2 triggers ferroptosis by disrupting USP38mediated SLC7A11 stabilization in chemoresistant gastric cancer

**DOI:** 10.1007/s00018-026-06345-4

**Published:** 2026-07-30

**Authors:** Runsha Xiao, Yan Mo, Jin Zhou, Yi Xu, Kuo Kang, Zhikang Chen, Yuanyuan Jiang, Chengmin Li, Xueping Feng, Zihua Chen, Si Chen, Chen Lai

**Affiliations:** 1 Department of General Surgery, Xiangya Hospital, Central South University, Changsha, 410008 China; 2 Hunan Key Laboratory of Precise Diagnosis and Treatment of Gastrointestinal Tumor, Xiangya Hospital, Central South University, Changsha, 410008 China; 3 National Clinical Research Center for Geriatric Diseases (Xiangya Hospital), Xiangya Hospital, Central South University, Changsha, 410008 China; 4 International Joint Research Center of Minimally Invasive Endoscopic Technology Equipment &Standardization, Xiangya Hospital, Central South University, Changsha, Hunan 410008 China; 5 Department of Clinical Laboratory, Xiangya Hospital, Central South University, Changsha, 410008 China; 6 Department of Pathology, Xiangya Hospital, Central South University, Changsha, 410008 China; 7 Department of Oncology, Institute of Medical Sciences, Xiangya Hospital, Central South University, Changsha, 410008 China; 8 Department of Ophthalmology, Xiangya Hospital, Central South University, Changsha, 410008 China; 9 Hunan Key Laboratory of Ophthalmology, Xiangya Hospital, Central South University, Changsha, 410008 China

**Keywords:** Metabolic reprogramming, Chemoresistance, Oxidative stress, Mitochondrial damage, Deubiquitinase

## Abstract

Aberrant expression of EphA2 (Ephrin receptor A2) in gastric cancer (GC) promotes chemoresistance by suppressing ferroptosis, yet the underlying mechanism remains poorly defined. In this study, we show that EphA2 confers resistance to platinum-based chemotherapy by suppressing ferroptosis through redox homeostasis regulation. Mechanistically, EphA2 directly phosphorylates and activates the deubiquitinase USP38, which in turn stabilizes SLC7A11 by preventing its proteasomal degradation. Elevated SLC7A11 sustains intracellular glutathione homeostasis, attenuates lipid peroxidation, and limits reactive oxygen species accumulation, thereby enabling GC cells to evade ferroptotic cell death under chemotherapeutic stress. Importantly, genetic or pharmacological disruption of the EphA2-USP38 interaction leads to SLC7A11 destabilization, excessive ROS accumulation, ferroptosis induction, and restoration of chemosensitivity. Together, these findings identify the EphA2-USP38/ROS axis as a key determinant of oxaliplatin resistance in GC and highlight a promising therapeutic target for overcoming chemotherapy resistance by reactivating ferroptosis.

**Supplementary Information:**

The online version contains supplementary material available at https://doi.org/10.1007/s00018-026-06345-4.

## Introduction

Gastric cancer (GC) is a primary malignant tumor that ranks fourth globally in cancer-related mortality, causing over 0.7 million fatalities annually [1]. Despite advancements in therapeutic techniques, neoadjuvant chemoradiotherapy followed by radical gastrectomy remains the primary treatment for GC patients [2]. Chemotherapy, predominantly based on fluorouracil and platinum compounds, remains a cornerstone of systemic treatment for GC, aiming to eradicate tumor cells and reduce the risks of metastasis and recurrence. However, the intrinsic and acquired heterogeneity of gastric tumors frequently undermines therapeutic efficacy, leading to chemoresistance, disease progression, and tumor relapse. These challenges substantially complicate clinical management and ultimately contribute to poor patient prognosis [3].

The receptor tyrosine kinase EphA2 is a 130-kDa transmembrane glycoprotein composed of 976 amino acids [4]. EphA2 is frequently overexpressed in tumor tissues, and it also crosstalks closely with hallmark oncogenes in GC (Table S1). Ferroptosis is a distinct form of regulated cell death characterized by the lethal accumulation of reactive oxygen species (ROS) dependent lipid peroxides [5–7]. Excessive ROS can attack polyunsaturated fatty acids (PUFAs) within cellular lipid membranes, triggering lipid peroxidation that compromises membrane integrity and ultimately leads to irreversible disruption of cellular homeostasis. Accumulating evidence indicates that ferroptosis is tightly regulated by tumor metabolic reprogramming [8, 9]. Under conditions of elevated oxidative stress, tumor cells may survive by activating compensatory antioxidant mechanisms, including the antioxidant defense system and the glutathione-dependent pathway, to prevent lethal lipid peroxidation [10–12]. Accordingly, previous studies have suggested that targeting metabolic vulnerabilities to induce ferroptosis represents a promising strategy to overcome chemoresistance in cancer therapy [13–15]. Therefore, elucidating the molecular mechanisms underlying chemoresistance is critical for addressing malignant phenotypes in GC.

In this study, we identified a previously unrecognized EphA2-USP38/ROS axis that suppresses ferroptosis and promotes chemoresistance through an ROS-dependent mechanism in GC.

## Materials and methods

### Cell culture and transfection

The cell lines used in this study, detailed in Table S2, were widely adopted in GC research. Lentiviral particles or plasmids (Genechem, China) were used to regulate EphA2 or USP38 expression according to the instructions. Prior to transfection, cells were seeded onto plates until they reached 60–80% confluence. Overexpression or small-interfering RNA (siRNA) plasmids (Genechem, China) were then transfected following the manufacturer’s instructions. After 72 h, puromycin (6 µg/ml) was used for 2–3 generations to select positive cells. GV248 was adopted for plasmids construction, and the detailed sequences were listed in Table S3.

### Public database and clinical samples analysis

The STAR-counts data (*n* = 375) along with the relevant clinical information for GC were obtained from The Cancer Genome Atlas (TCGA) database (portal.gdc.cancer.gov). The patients (*n* = 125) enrolled in the study underwent postoperative pathological diagnosis according to the Chinese Society of Clinical Oncology (CSCO) Clinical Guidelines for Gastric Cancer [16]. Clinical information was collected for survival analysis. Our study was approved by the ethics committee, and all patients were provided with informed consent. Table S4 provided the general information of patients.

### Western blotting analysis and Immunoprecipitation

Cells were harvested and boiled in 1 × SDS loading buffer, followed by SDS-PAGE separation using Mini-PROTEAN^®^ Tetra (#1658004, Bio-Rad, US). For co-immunoprecipitation, cells were lysed in IP lysis buffer supplemented with 1 × protease inhibitor cocktail. Cell lysates were pre-cleared with protein A/G magnetic beads (#HY-K0202, MCE, US) for 1 h before they were immunoprecipitated with antibody. The following reagents were used in study: EphA2 (#sc-398832, 1:1000, Santa Cruz, USA), USP38 (#17767-1-AP, 1:1000, Proteintech, USA), SLC7A11 (#26864-1-AP, 1:1000, Proteintech, USA), 4-HNE (#bs-6313R, 1:1000, Bioss, China), HRP-conjugated secondary antibodies against mouse (#SA00001-1, 1:5000, Proteintech, USA) and rabbit (#SA00001-2, 1:5000, Proteintech, USA).

### Immunohistochemistry (IHC)

All samples were formalin-fixed, embedded in paraffin, and then sectioned into 4 μm slices. After deparaffinization, antigen retrieval, quenching endogenous peroxidase activity and blocking nonspecific binding. The sections were subsequently incubated overnight at 4 °C with antibodies against EphA2 (#66736-1-Ig, 1:200, Proteintech, USA), SLC7A11 (#26864-1-AP, 1:200, Proteintech, USA), and 4-HNE (#bs-6313R, 1:200, Bioss, China). Following this, the sections were incubated with a horseradish peroxidase-conjugated secondary antibody (#RGAU011, 1:3000, Proteintech, USA). Then visualized with diaminobenzidine and counterstained with hematoxylin before observation. The evaluation of immunohistochemical staining was based on a previous study [17].

### Multiplex immunohistochemistry

Co-staining of EphA2 and SLC7A11 was performed using a Seven-color multiple immunofluorescence Kit (#RC0086-67R, Recordbio, China) based on the tyramide signal amplification (TSA) technology. All slides were visualized on the Quantitative Pathology Imaging System (Vectra Polaris, AKOYA, USA).

### Cell counting kit-8 (CCK-8)

Reagent CCK-8 (#521942, Biosharp, China) was used to evaluate the viability of cells. The data of absorbance at 490 nm in each well were measured for calculation.

### Transwell assay

After properly plated into the chamber, cells were cultured in serum-free medium for 24 h. Cells were gently wiped off from the upper chambers, and crystal violet was used for staining.

### RNA extraction and qRT-PCR

TRIzol reagent (#15596026CN, Invitrogen™, USA) was used for total RNA extraction. The PrimeScript™ RT reagent Kit (#RR037A, Takara, Japan) was used for reverse transcription. QPCR Master Mix (#18526ES, YEASEN, China) was used for qPCR. DNase was used for RNA purification of RNA extracted from transfected cells. GAPDH was used as the reference gene. The detailed sequences were listed in Table S5.

### Cysteine (Cys) and glutathione (GSH) assay

The intracellular level of cysteine was measured using a cysteine (Cys) colorimetric assay kit (#E-BC-K352-M, Elabscience, China). Intracellular level of total glutathione/oxidized glutathione (T-GSH/GSSG) in cells was detected using a total glutathione (T-GSH)/oxidized glutathione (GSSG) colorimetric assay kit (#E-BC-K097-M, Elabscience, China). The specific operation method followed the manufacturer’s instructions.

### Reactive oxygen species (ROS) and lipid peroxide (LPO) assay

BODIPY™ 581/591 C11 (#D3861, Invitrogen™, USA) was used for lipid-ROS staining. Fluorescence intensity of the samples was determined using flow cytometry. The fluorescent dyes used in confocal microscopy (CLMS) were Mito-Tracker (#C1048, Beyotime, China) and BODIPY™ 581/591 C11 (#D3861, Invitrogen™, USA). The concentration of LPO in cells was detected using Lipid Peroxide (LPO) Colorimetric Assay Kit (#E-BC-K176-M, Elabscience, China). The specific operation method followed the manufacturer’s instructions.

### Mitochondrial membrane potential imaging

JC-1 staining (#M34152, Invitrogen™, USA) was used to observe the mitochondrial potential. Cells were previously well grown in vessel, then 10 mg/ml JC-1 was added into the medium and incubated at 37 °C in the dark for 10 min to label the mitochondrion. Normal mitochondrial potential showed red fluorescence, and damaged mitochondrial potential showed green fluorescence.

### Protein degradation assay

Cells were seeded onto plates, then cycloheximide (# C7698, 40 µM, Sigma, USA) was added into each well at pre-set time points. MG-132 (#A11043, 10 µM, AdooQ, USA) was used to inhibit proteasome-mediated protein degradation.

### *In vivo* mouse cells-derived xenograft (CDX) model

Well-grown cells were resuspended to obtain single-cell suspension, then subcutaneously inoculated in the right forelimb armpit of 4-week-old BALB/c female nude mice. After the tumor grew up to approximately 50 mm^3^, mice were used as the CDX model and grouped for different processing. All reagents used for in vivo injections were endotoxin-free (< 0.1 EU/µg). The animal procedures were approved by the Animal Ethics and welfare Committee, at Central South University (No. 2023030512). All animal studies were carried out using the Institutional Animal Care and Use Committee (IACUC) approved procedures.

### Molecular docking

The molecular docking model was predicted by Cluspro 2.0 Server (https://cluspro.bu.edu) [18–21]. The PyMOL Molecular Graphics System (http://www.pymol.org/) was adopted to generate the structural illustration based on the sequence of amino acid.

### Statistical analysis

All experiments were performed with at least three independent biological replicates (*n* = 3). GraphPad Prism 9.0, SPSS 24.0, and R studio software (version 4.2.1) were used for statistical analyses. Data were expressed as mean ± SD. Normality of data distribution was assessed using the Shapiro-Wilk test, and homogeneity of variances was assessed using Levene’s test. The comparisons between two groups were analyzed using two-tailed unpaired Student’s t-test, while comparisons among three or more groups were analyzed using one-way analysis of variance (ANOVA) followed by Tukey’s post hoc test for multiple comparisons. Kaplan-Meier survival curves were compared using the log-rank (Mantel-Cox) test. The differences were considered statistically significant with a *P*-value < 0.05. Significance levels are indicated as follows: * *P* < 0.05, ** *P* < 0.01, *** *P* < 0.001, **** *P* < 0.0001, while “ns” represents no significant difference.

## Results

### High EphA2 expression is associated with poor prognosis and oxidative stress-related pathways in GC

**Fig. 1:**
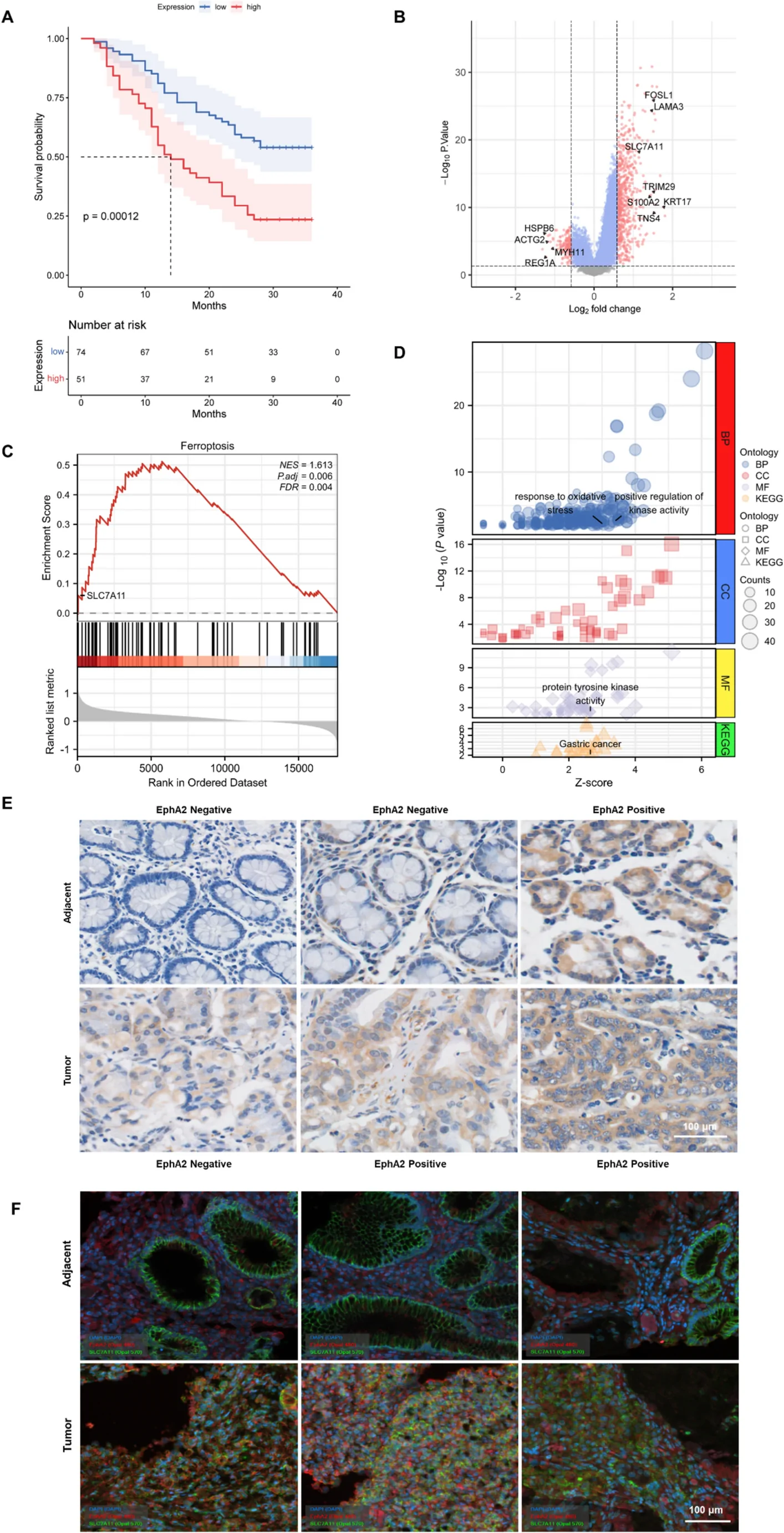
High expression level of EphA2 in GC is associated with poor prognosis and oxidative stress. (**A**) KM survival curve based on the clinical information from 125 GC patients, cut-off selection: median expression value. (**B**) A volcano plot displayed the magnitude of gene expression changes related to EphA2. Red points represent genes whose FC and P-value both meet the thresholds, indicating that the change in gene expression is both significant and large, *n* = 375. (**C**) GSEA enrichment analysis revealed upregulation of ferroptosis pathway expression, *n* = 375. (**D**) Functional enrichment analysis based on differential expression of EphA2 related genes in GC, *n* = 375. (**E**) IHC staining results showed high expression level of EphA2 in tumor and paired adjacent tissue sections. Scale bar: 100 μm. (**F**) Co-staining of EphA2 (red), SLC7A11 (green) and DAPI (blue) in sections based on TSA system. Scale bar: 100 μm

High-throughput sequencing data from the TCGA database revealed that EphA2 expression was significantly elevated in gastric cancer (GC) tissues (Fig. S1A, *n* = 375). This observation was further validated by immunohistochemical (IHC) analysis of tumor samples from 54 GC patients (*P* < 0.001, Fig. S1B). Similarly, Western blot analysis in GC cell lines also demonstrated the statistical significance of this difference (*P* < 0.0001, Fig. S1C and D). Kaplan-Meier survival analysis demonstrated a significant difference in overall survival between patients with high and low EphA2 expression (Fig. 1A), with patients exhibiting high EphA2 expression showing markedly poorer prognosis (*P* < 0.01). Univariate Cox regression analysis further revealed that high EphA2 expression was significantly associated with poor overall survival (HR = 2.38, *P* < 0.001).

Differentially expressed genes associated with EphA2 expression were visualized using a volcano plot (Fig. 1B). Notably, SLC7A11, a key regulator of ferroptosis [9], was identified among the significantly upregulated genes. Gene set enrichment analysis (GSEA) further indicated that genes upregulated in the high EphA2 expression group were significantly enriched in the ferroptosis pathway (Fig. 1C). Functional enrichment analyses, including Gene Ontology (GO) and KEGG pathway analyses (Fig. 1D), revealed that EphA2-associated differentially expressed genes were predominantly involved in oxidative stress response and protein tyrosine kinase activity. Multiplex immunohistochemistry on tissue sections from clinical GC patients (Fig. 1F) showed that the relative fluorescence intensity of EphA2 (red) and SLC7A11 (green) was higher in GC tissues (tumor) than in adjacent normal tissues (adjacent). Moreover, SLC7A11 expression in GC tissues, particularly those with poor differentiation and heterogeneity, appeared to correlate with EphA2 expression. Furthermore, a comprehensive clinical evaluation incorporating chemotherapy response, computed tomography (CT) imaging, tumor biomarker levels, and patient clinical symptoms revealed that high EphA2 expression was significantly associated with chemoresistance in GC patients (Table S4).

### EphA2 maintains the morphological stability of mitochondria and cell viability of GC cells under oxidative stress

**Fig. 2:**
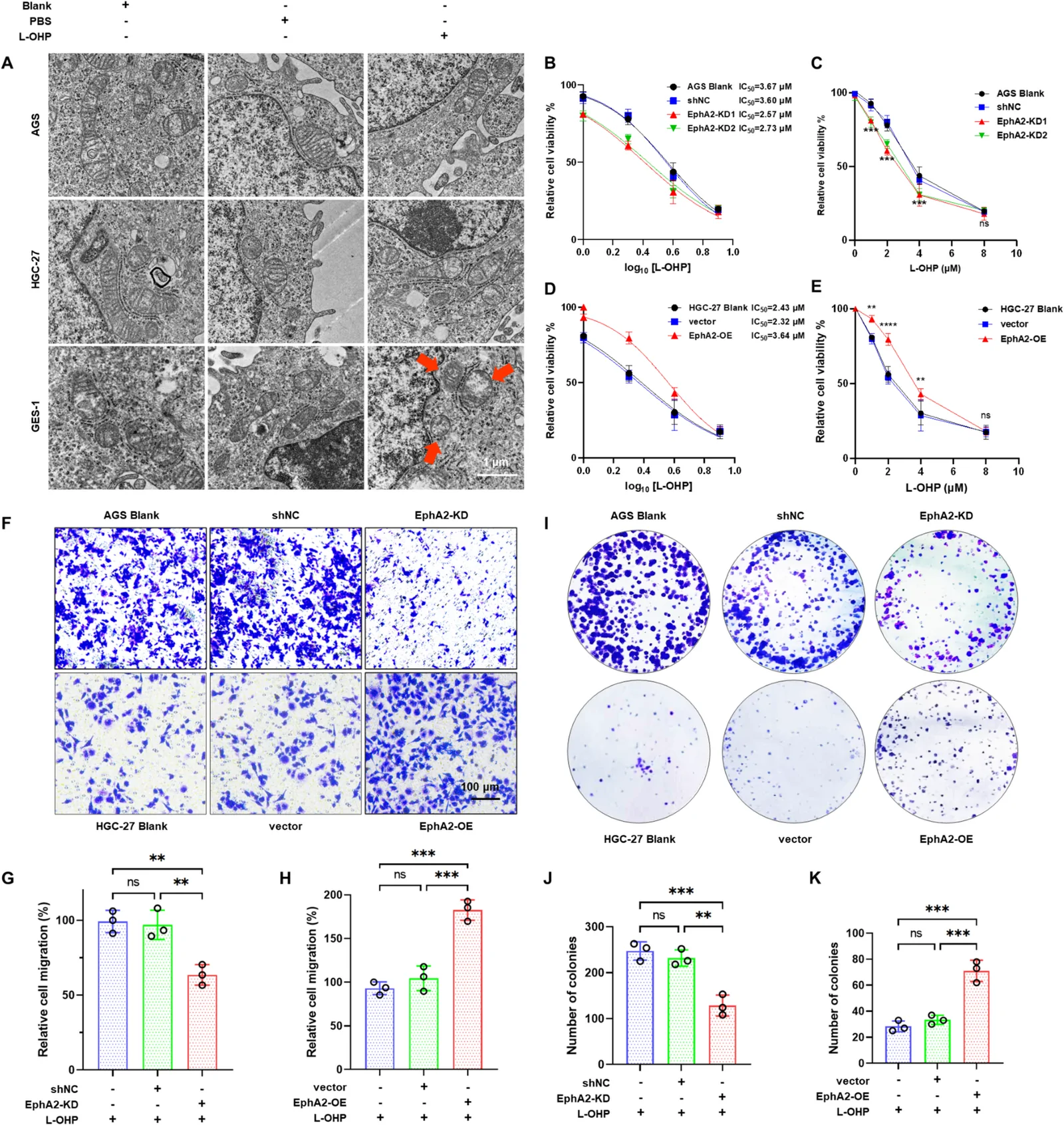
EphA2 regulates the cell viability of GC cells under oxidative stress induced by L-OHP. (**A**) Transmission electron microscope (TEM) images of morphological changes of cell mitochondrion under oxaliplatin induced oxidative stress. AGS and HGC-27 cells were less affected than GES-1 cells in density enhancement and degree of mitochondrial shrinkage. Scale bar: 1 μm. (**B**-**C**) Measurement of cell viability via CCK-8 assay under 24 h L-OHP treatment at different concentrations. The cell viability of AGS cells was significantly inhibited in EphA2-KD groups (*P* < 0.001, the best-fit IC50 of L-OHP for AGS cells was 3.67 µM), *n* = 3. (**D**-**E**) The relative cell viability of EphA2-OE group was prominently promoted in HGC-27 cells (*P* < 0.01, the best-fit IC50 concentration of L-OHP to HGC-27 cells was 2.43 µM). (**F**-**H**) Transwell assay of GC cells treated with L-OHP 24 h at IC50 in different groups. Scale bar: 100 μm. Migration ability decreased in EphA2-KD group of AGS cells, while an elevated migration ability was observed in EphA2-OE group of HGC-27, *n* = 3. (**I**-**K**) Results of colony formation assay, together with statistical bar charts of clone number, of GC cells after treated with L-OHP for 24 h in different groups, *n* = 3. Cell proliferation ability inhibited in EphA2-KD group of AGS cells, while promoted cell proliferation ability was noticed in EphA2-OE group of HGC-27 cells. The asterisks symbol indicates significant differences between groups: **P < 0.01, ***P < 0.001, ****P < 0.0001, while “ns” represents no significant difference

Ferroptosis and oxidative stress are tightly interconnected biological processes that critically influence cellular viability, including the development of drug resistance [22, 23]. Oxaliplatin (L-OHP), a platinum-based anticancer drug commonly used in the chemotherapy of GC, has been reported to induce oxidative stress [24]. Consistently, flow cytometry analysis demonstrated that oxaliplatin treatment significantly increased intracellular reactive oxygen species (ROS) levels (*P* < 0.001, Fig. S2). Transmission electron microscopy (TEM) further revealed distinct mitochondrial ultrastructural alterations following oxaliplatin exposure. As shown in Fig. 2A, mitochondrial damage was markedly less pronounced in GC cells (AGS and HGC-27) than in normal gastric epithelial cells (GES-1), characterized by reduced mitochondrial shrinkage and preserved mitochondrial density. These observations suggest that EphA2 may contribute to maintaining mitochondrial structural integrity in GC cells under oxaliplatin-induced oxidative stress.

To evaluate the effect of EphA2 on the viability of GC cells under L-OHP treatment, CCK-8 assays were conducted. The results indicated that as the concentration of L-OHP increased, the knockdown of EphA2 in AGS cells initially led to a significant reduction in cell viability at various concentrations of L-OHP, compared to control groups (*P* < 0.001), with a best-fit IC_50_ value of 3.67 µM for AGS cells (Fig. 2B and C). However, at higher concentrations of L-OHP, the disparity in cell viability between EphA2-knockdown AGS cells and controls diminished, ultimately showing no significant difference. In contrast, the overexpression of EphA2 in HGC-27 cells significantly enhanced cell viability compared to control groups (*P* < 0.001), with a best fit IC_50_ value of 2.43 µM for HGC-27 cells (Fig. 2D-E). And the time-dependent CCK-8 assay of cell viability in AGS cells suggested that the reasonable therapeutic time window at the half-maximal inhibitory concentration (IC_50_) is between 18.17 and 23.53 h (95% CI, Fig. S3). Transwell assay was conducted to evaluate the effect of EphA2 on the migration ability under L-OHP treatment (Fig. 2F). The results indicated that EphA2 knockdown in AGS cells decreased cell migration (Fig. 2G) treated by L-OHP (IC_50_ concentration), while EphA2 overexpression in HGC-27 promoted migration (Fig. 2H). Furthermore, a colony formation assay was conducted to assess the impact of EphA2 on cell proliferation under L-OHP treatment (Fig. 2I). The results showed that cell proliferation ability was inhibited after EphA2 knockdown in AGS cells (Fig. 2J) treated with L-OHP (IC_50_ concentration), compared to a promoted cell proliferation ability after EphA2 overexpression in HGC-27 cells (Fig. 2K). Collectively these findings demonstrate that high EphA2 expression helps reducing susceptibility to ferroptosis in GC cells while maintaining their cell activity under L-OHP treatment.

### EphA2 participates in the regulation of lipid-ROS dependent oxidative stress and mitochondrial disorder

**Fig. 3:**
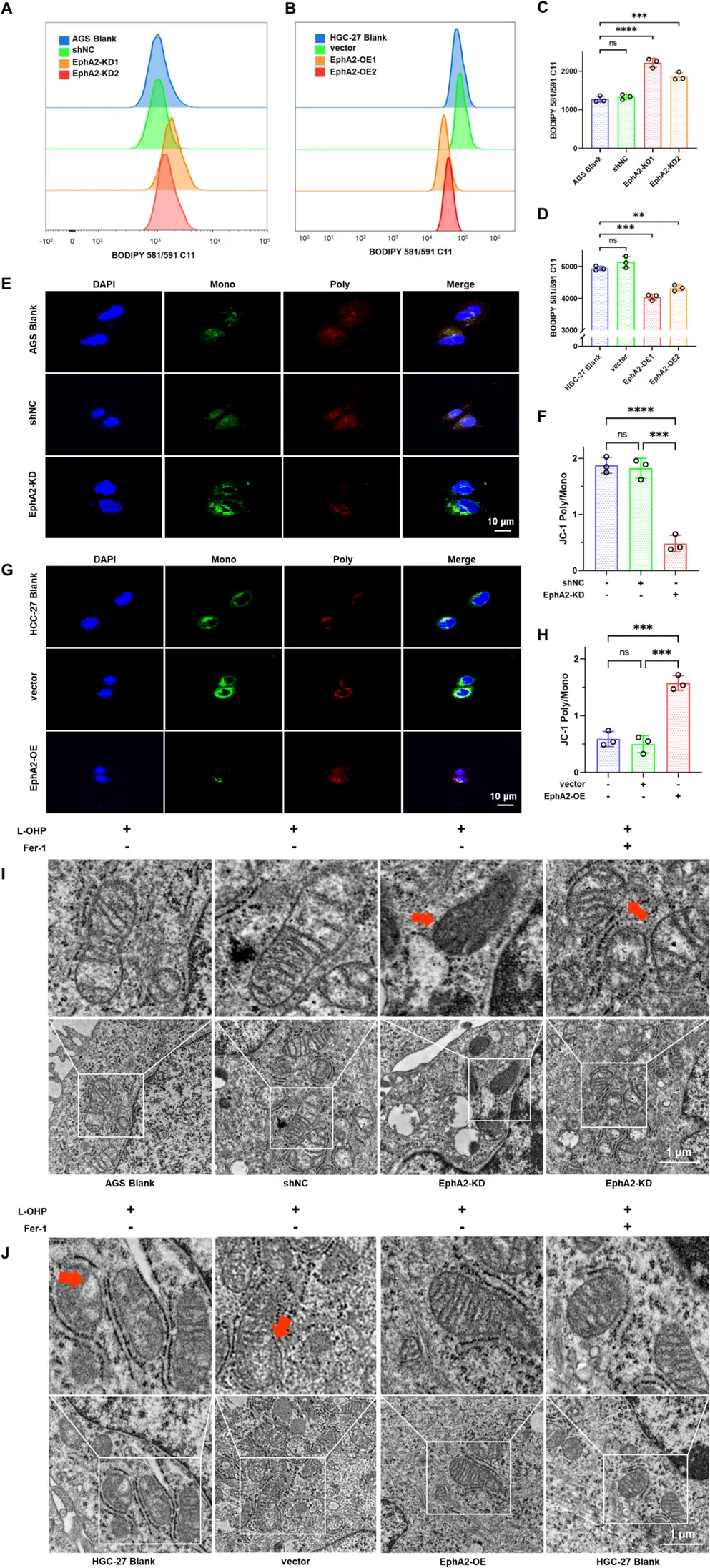
EphA2 involved in regulating lipid-ROS dependent ferroptosis resistance in GC cells. (**A**-**B**) Flow cytometry results of lipid-ROS using BODIPY™ 581/591 C11 dye in GC cells after L-OHP treatment (IC50 for 24 h). (**C**) The lipid-ROS accumulation was found significantly increased in EphA2-KD group of AGS cells, (**D**) compared with the inhibited accumulation of lipid-ROS in EphA2-OE groups of HGC-27 cells, *n* = 3. (**E**-**F**) CLSM images of mitochondrial membrane potential in AGS cells using JC-1 dye and comparison of Poly/Mono value of JC-1 after L-OHP treatment, *n* = 3. Scale bar: 10 μm. (**G**-**H**) CLSM images and Poly/Mono comparison in HGC-27 cells, *n* = 3. Scale bar: 10 μm. (**I**) TEM images of AGS cells pre-treated with L-OHP for 24 h at concentration of IC50. Disrupted mitochondrial membrane appeared in EphA2-KD group of AGS cells, while the utilization of Fer-1 reversed this damage. Scale bar: 1 μm. (**J**) TEM images of HGC-27 cells after L-OHP treatment (IC50 for 24 h). EphA2-OE group showed more integral and distinct mitochondrial structure compared to the control groups. The damage on mitochondrial membrane could be inhibited after the utilization of Fer-1. The asterisks symbol indicates significant differences between groups: **P < 0.01, ***P < 0.001, ****P < 0.0001, while “ns” represents no significant difference. Scale bar: 1 μm

Lipid reactive oxygen species (lipid ROS) are lethal byproducts of oxidative stress and have been extensively investigated in the context of antioxidant defense and ferroptosis [25]. Flow cytometry showed EphA2 knockdown significantly increased lipid ROS levels in AGS cells (Fig. 3A and C), whereas EphA2 overexpression significantly inhibited the accumulation of lipid ROS in HGC-27 cells (Fig. 3B and D). The CLMS images (Fig. S4) showed that oxaliplatin treatment significantly increased mitochondrial lipid ROS levels in GC cells (*P* < 0.01), while inhibition of EphA2 further amplified this effect (*P* < 0.0001).

Mitochondria play a central role in the initiation and execution of ferroptosis [26], and undergo characteristic ultrastructural alterations during this process, including increased membrane density, loss of mitochondrial cristae, and a shrunken morphology [27]. To further evaluate mitochondrial functional integrity, JC-1 staining was employed to assess mitochondrial membrane potential. CLSM analysis revealed that EphA2 knockdown significantly increased green fluorescence intensity in AGS cells (Fig. 3E and F), indicative of mitochondrial membrane depolarization and enhanced mitochondrial damage under oxaliplatin-induced oxidative stress. Conversely, EphA2 overexpression markedly reduced green fluorescence in HGC-27 cells (Fig. 3G and H), further supporting a protective role of EphA2 against ROS-dependent mitochondrial dysfunction.

Transmission electron microscopy (TEM) was used to capture these morphological changes induced by L-OHP. As shown in images (Fig. 3I), EphA2 knockdown resulted in severe mitochondrial damage, characterized by disrupted membrane integrity, increased mitochondrial density, and marked shrinkage, compared to control groups. Importantly, these mitochondrial abnormalities were largely reversed by treatment with ferrostatin-1 (Fer-1), a synthetic ferroptosis inhibitor, indicating that the observed mitochondrial damage was ferroptosis-dependent. In contrast, EphA2 overexpression in HGC-27 cells was associated with markedly preserved mitochondrial ultrastructure following oxaliplatin treatment, with substantially less mitochondrial damage observed compared to control groups (Fig. 3J). Notably, treatment with Fer-1 in the HGC-27 control group similarly prevented oxaliplatin-induced mitochondrial injury.

### EphA2 enhanced the stability of SLC7A11 by phosphorylating deubiquitinase USP38

**Fig. 4:**
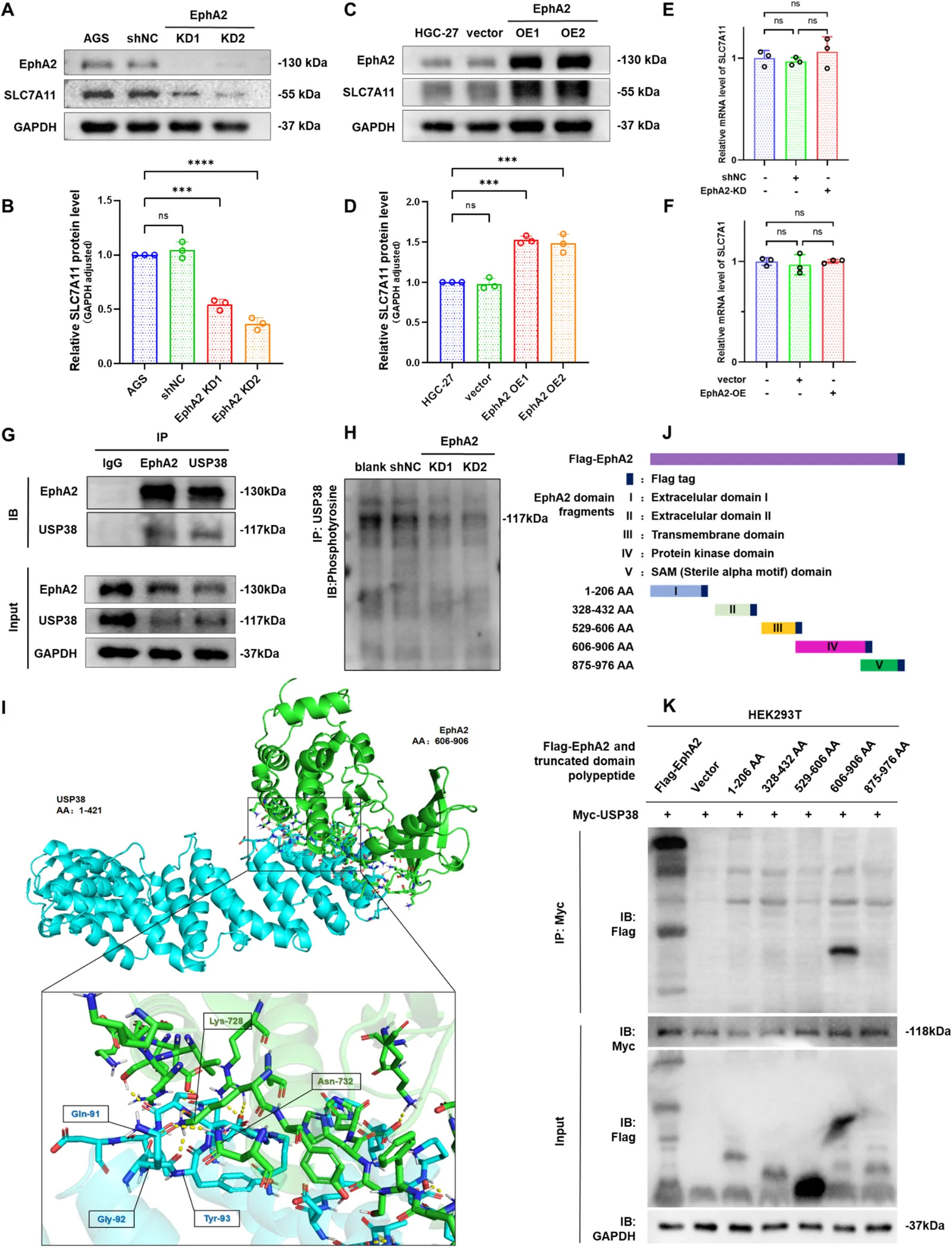
EphA2 enhanced the stability of SLC7A11 through phosphorylating deubiquitinase USP38. (**A-B**) Western blotting (WB) results indicated that knockdown EphA2 decreased SLC7A11 protein level in AGS cells, (**C-D**) while overexpression of EphA2 increased SLC7A11 protein level in HGC-27 cells, *n* = 3. (**E-F**) The relative mRNA level of SLC7A11 suggested that EphA2 does not transcriptionally regulate it in AGS or HGC-27 cells, *n* = 3. (**G**) Western blotting results after Immunoprecipitation (IP) suggested a protein-protein interaction (PPI) between EphA2 and USP38. (**H**) The tyrosine phosphorylation level of USP38 after the IP of SLC7A11 in different groups of AGS cells. (**I**) Predicted docking area of EphA2 (green) and USP38 (blue) using Cluspro 2.0 Server (https://cluspro.bu.edu). Pymol software was used to display the highlighted sites (Gln: glutamine, Gly: glycine, Tyr: tyrosine, Lys: lysine, Asn: aspartic acid, numbers present amino acid sequence sites). (**J**) Schematic diagram of EphA2 protein full-length and structural domain truncated plasmid design. (**K**) After infecting HEK293T cells with truncated plasmids and after the co-IP procedure, the WB results indicated the EphA2 protein kinase domain (amino acids 606–906) was probably responsible for the molecular binding between EphA2 and USP38. The symbol ns represents no significant difference. The asterisks symbol indicates significant differences between groups: ***P < 0.001, ****P < 0.0001, while “ns” represents no significant difference. The “ns” symbol represents no significant difference

Metabolic regulation plays a pivotal role in cellular resistance to oxidative stress and ferroptosis [13, 28]. Western blotting found that EphA2 could regulate the level of protein SLC7A11 (Fig. 4A and C). The knockdown of EphA2 significantly decreased SLC7A11 protein level in AGS cells (Fig. 4B, *P* < 0.001). While overexpression of EphA2 prominently increased SLC7A11 protein level in HGC-27 cells (Fig. 4D, *P* < 0.001). Furthermore, qPCR analysis conducted on AGS cells demonstrated that there was no statistically significant difference in the mRNA quantification of SLC7A11 between the groups with and without EphA2 knockdown. (Fig. 4E). Similarly, the overexpression of EphA2 in HGC-27 cells did not significantly alter the mRNA quantification of SLC7A11 (Fig. 4F). These results indicated that EphA2 may regulate the level of SLC7A11 at the post-transcriptional level. To identify potential EphA2-interacting proteins involved in this regulation, immunoprecipitation (IP) was performed using an anti-EphA2 antibody (#sc-398832, Santa Cruz, USA) to identify candidate interacting proteins followed by mass spectrometry analysis. Among the candidates identified, the deubiquitinase USP38 emerged as a prominent interacting protein (Table S6). Subsequent co-immunoprecipitation and Western blot analyses confirmed a direct protein-protein interaction between EphA2 and USP38 (Fig. 4G). In addition, Western blot analysis further indicated a physical interaction between USP38 and SLC7A11, suggesting that USP38 may function as a molecular link between EphA2 and SLC7A11 stability.

Given that EphA2 is a receptor tyrosine kinase, we next examined whether EphA2 regulates USP38 via tyrosine phosphorylation. Immunoblotting with a pan-phosphotyrosine antibody (#T91162, Abmart, China) revealed a marked reduction in USP38 tyrosine phosphorylation levels following EphA2 knockdown (Fig. 4H), indicating that EphA2 promotes USP38 phosphorylation. To explore the structural basis of this interaction, protein-protein docking analysis was performed using ClusPro 2.0 (https://cluspro.bu.edu), which predicted a potential interaction interface between EphA2 and USP38 (Fig. 4I).

To further map the EphA2 domain responsible for binding USP38, a series of truncated EphA2 constructs were generated based on its annotated domain structure and full-length amino acid sequence (UniProt) (Fig. 4J). HEK293T cells were used to stably express Myc-tagged USP38. Then, co-immunoprecipitation assays demonstrated that the protein kinase domain of EphA2 (amino acids 606–906) was essential for mediating the interaction with USP38 (Fig. 4K).

**Fig. 5:**
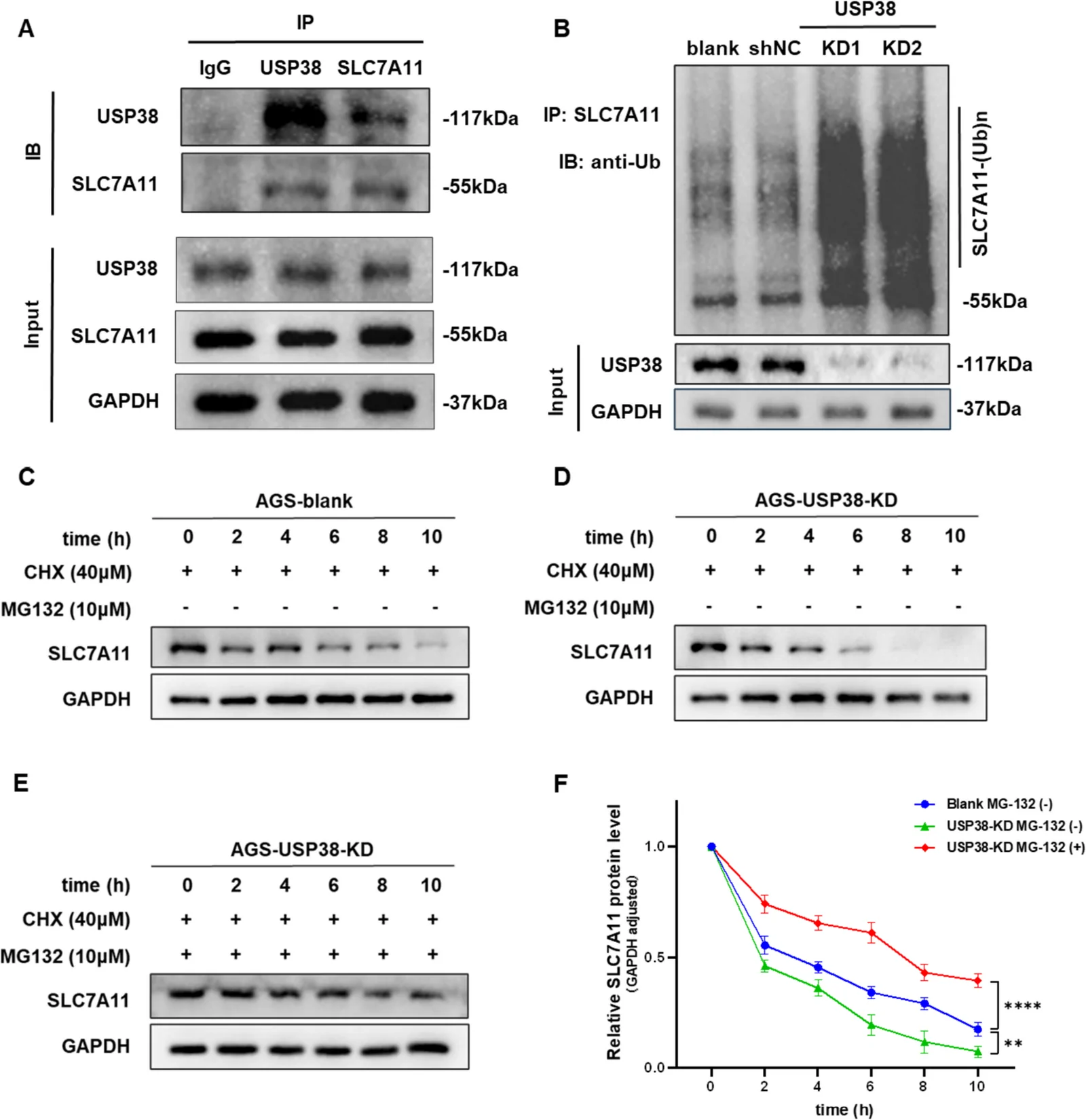
USP38 regulates the stability of SLC7A11 protein through the ubiquitin proteasome system pathway. (**A**) Immunoprecipitation (IP) results indicated interaction between protein USP38 and SLC7A11. (**B**) WB results of ubiquitination level of SLC7A11 using ubiquitin antibody (P4D1, Santa Cruz, USA) in different groups of AGS cells. (**C-E**) WB results of SLC7A11 protein level in different groups of cells. CHX (40 µM) and/or MG-132 (10 µM) were pre-added to different groups of cells. (**F**) The line chart of SLC7A11 protein degradation rate indicated that the knockdown of USP38 could significantly accelerate the degradation of SLC7A11, while MG-132 could rescue this effect, *n* = 3. The asterisks symbol indicates significant differences between groups: **P < 0.01, ****P < 0.0001, while “ns” represents no significant difference

USP38 belongs to the deubiquitinase (DUB) family, prompting us to investigate whether USP38 regulates SLC7A11 stability through deubiquitination. Co-immunoprecipitation assays confirmed a direct interaction between USP38 and SLC7A11 (Fig. 5A). AGS cells expressing control shRNA or USP38 shRNA were treated with MG-132 (10 µM) for 6 h prior to harvest. Then, ubiquitination assays using an anti-ubiquitin antibody (P4D1, Santa Cruz, USA) following SLC7A11 immunoprecipitation revealed that USP38 knockdown significantly increased the ubiquitination level of SLC7A11 (Fig. 5B). Next, the rate of SLC7A11 protein degradation were analyzed by Western blot (Fig. 5C-E). And the line chart demonstrated that the knockdown of USP38 significantly accelerated SLC7A11 degradation (Fig. 5F, *P* < 0.01), whereas treatment with the proteasome inhibitor MG-132 (10 µM) prominently attenuated the degradation SLC7A11 from the hydrolysis of proteasome (Fig. 5F, *P* < 0.0001). These assays indicated that USP38 participates in regulating the degradation of SLC7A11 protein through the proteasome pathway.

### Loss of EphA2 promotes ferroptotic vulnerability in GC cells

**Fig. 6:**
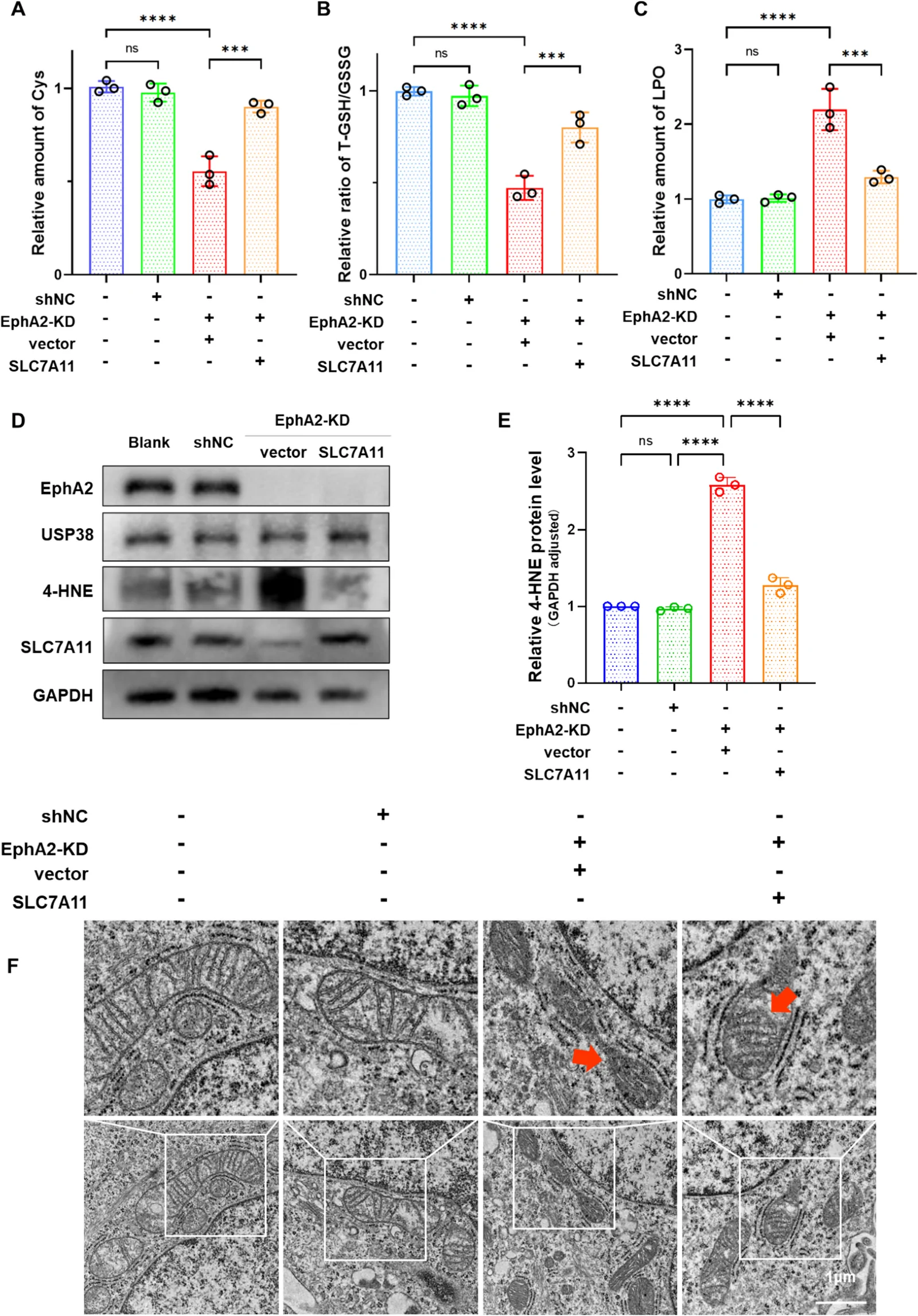
Rescue the effect of inhibiting EphA2 on attenuating the endurance to oxidative stress in GC cells. After a 24-hour pre-treatment of L-OHP in AGS cells, the following experiments were conducted: (**A**) The relative amount of intracellular cysteine (Cys). A decreased level of Cys was found in the EphA2-KD group, and this decrease was reversed upon the transfection of SLC7A11 plasmid, *n* = 3. (**B**) The relative amount of intracellular T-GSH/GSSG. A reduction of the T-GSH/GSSG was found in EphA2-KD group. It was rescued by the transfection of SLC7A11 plasmid, *n* = 3. (**C**) The relative amount of intracellular lipid peroxide (LPO). More accumulation of LPO was detected in EphA2-KD group, which was also remarkably mitigated by SLC7A11 plasmids, *n* = 3. (**D**) Western blotting results of 4-HNE in AGS cells. (**E**) The results revealed that the level of 4-HNE was significantly increased in EphA2-KD group, *n* = 3. (**F**) Transmission electron microscopy (TEM) images indicated a more severe degree of morphological damage of mitochondria in EphA2-KD group, with shrinkage and increased density of mitochondria. And it was rescued by the transfection of SLC7A11 plasmid. The asterisks symbol indicates significant differences between groups: ***P < 0.001, ****P < 0.0001, while “ns” represents no significant difference. Scale bar: 1 μm

After 24 h of L-OHP treatment at the IC_50_ concentration in AGS cells, intracellular cysteine levels were quantified (Fig. 6A). As shown in Fig. 6A, cysteine levels were markedly reduced in the EphA2 knockdown (EphA2-KD) group, whereas ectopic expression of SLC7A11 effectively restored intracellular cysteine levels. In cellular anti-oxidative metabolism, cysteine provides the sulfhydryl group (-SH) which is crucial for the formation of the reduced form of glutathione (GSH). Consistently, GSH assay (Fig. 6B) revealed a declined ratio of total glutathione/oxidized glutathione (T-GSH/GSSG) in EphA2-KD group, indicating compromised redox homeostasis and increased susceptibility to oxidative stress. Further analysis demonstrated a pronounced accumulation of lipid peroxidation products in EphA2-KD AGS cells. As shown in Fig. 6C, lipid peroxides (LPO) were significantly elevated following EphA2 knockdown. This further confirmed there was an increased accumulation of 4-hydroxynonenal (4-HNE) in the EphA2-KD group. Western blot analysis revealed that the enforced SLC7A11 expression could significantly suppress 4-HNE accumulation in AGS cells (Fig. 6D and E, *P* < 0.0001).

TEM analysis revealed that EphA2 inhibition induced marked mitochondrial abnormalities, including increased membrane density and loss of mitochondrial cristae (Fig. 6F), which are characteristic ultrastructural features of ferroptosis. Importantly, these mitochondrial defects were largely reversed by SLC7A11 overexpression, indicating that EphA2 maintains ferroptosis resistance through the SLC7A11-dependent antioxidant pathway. In addition, WB analysis of pH2AX (a DNA damage marker protein, Fig. S5A and B) revealed that EphA2 knockdown significantly exacerbated L-OHP-induced DNA double-strand damage (*P* < 0.05). Additional nucleic acid damage analysis (Fig. S5C and D) further supported this finding by visually demonstrating the DNA tails, which serve as a direct indicator of DNA damage (*P* < 0.0001).

### *In vivo* validation of EphA2 knockdown sensitizes GC to chemotherapy

**Fig. 7:**
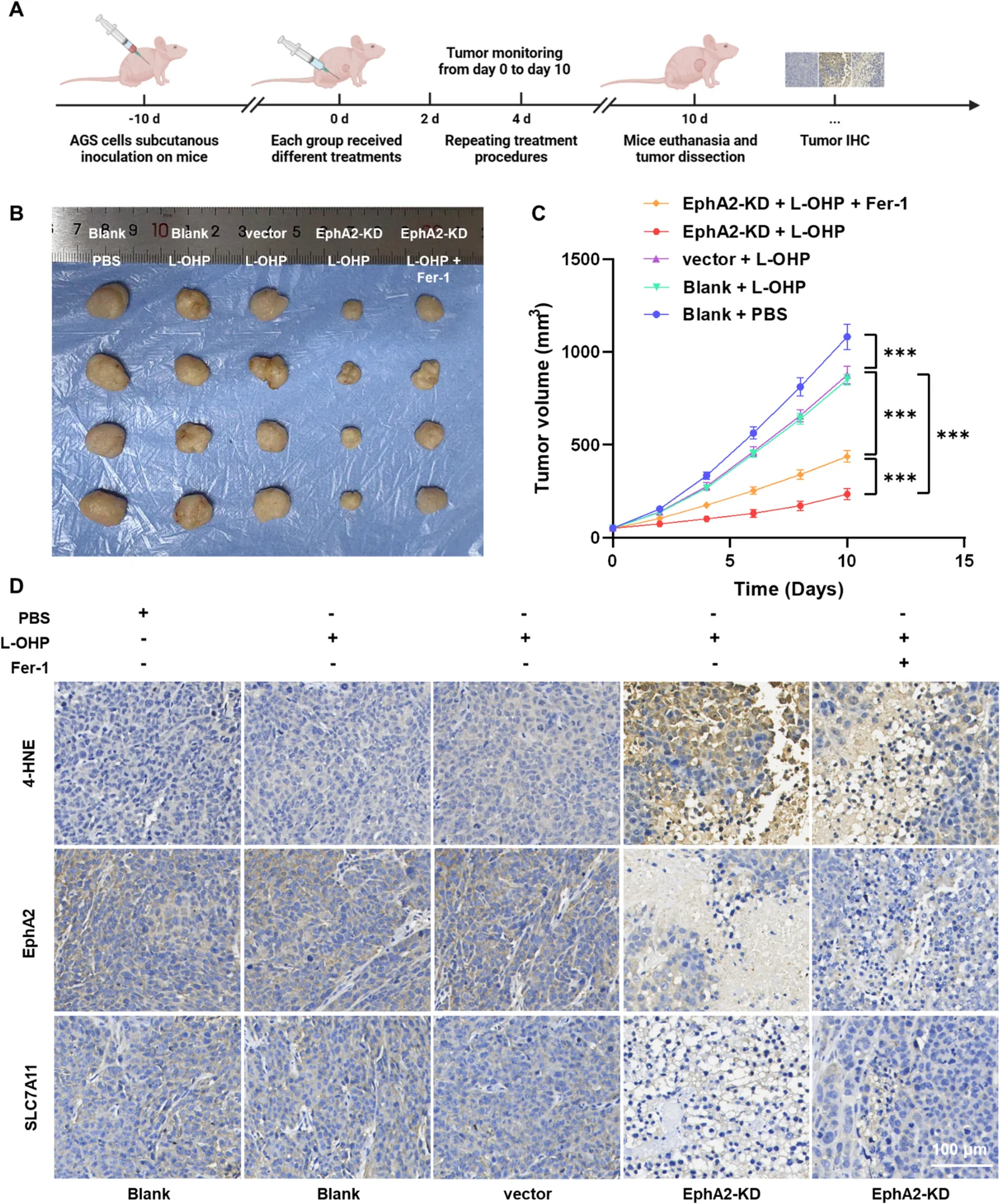
In vivo experiments of EphA2’s effect on GC chemotherapy resistance. (**A**) The timeline of establishment of GC CDX-model and treatment procedures. (**B**) Image of dissected tumors from different groups of mice, *n* = 4. The tumor growth was suppressed by L-OHP treatment in EphA2-KD group. (**C**) The curve of tumor volume (mm3) in different groups, *n* = 4. (**D**) IHC staining of 4-HNE, EphA2 and SLC7A11 in subcutaneous tumor tissue dissected from different groups of mice. A decreased level of SLC7A11 and accumulation of 4-HNE was found in EphA2-KD group, which could be rescued by Fer-1. The asterisks symbol indicates significant differences between groups (*** *P* < 0.001). Scale bar: 100 μm

To further evaluate the role of EphA2 in regulating chemotherapy resistance in vivo, BALB/c nude mice were maintained under specific pathogen-free (SPF) conditions and randomly assigned to five experimental groups, including two blank control groups, one negative vector control group, and two EphA2 knockdown groups. The schematic timeline is illustrated in Fig. 7A. Mice received intraperitoneal injections of PBS (100 µL, i.p.), L-OHP (10 mg/kg, i.p.), or L-OHP (10 mg/kg, i.p.) combined with the antioxidant ferrostatin-1 (Fer-1, 5 mg/kg). At the experimental endpoint (after 10 days of treatment), mice were first anesthetized with CO₂ inhalation, then euthanized by cervical dislocation. Then, tumors were excised from euthanized mice. Representative images of dissected tumors (Fig. 7B) and curves of tumor volume (Fig. 7C) demonstrated that EphA2 knockdown significantly enhanced the antitumor efficacy of L-OHP treatment, resulting in markedly suppressed tumor growth compared to control groups. Notably, co-administration of Fer-1 partially reversed the tumor-suppressive effect observed in the EphA2-KD + L-OHP group, indicating that ferroptosis contributed to the observed chemosensitization. IHC analysis further revealed that EphA2 knockdown was associated with reduced SLC7A11 expression and increased accumulation of 4-HNE within tumor tissues, accompanied by more severe histopathological damage (Fig. 7D). Importantly, these effects were substantially attenuated by Fer-1 treatment, further supporting the notion that EphA2 promotes chemoresistance in GC by suppressing ferroptosis in vivo.

**Fig. 8:**
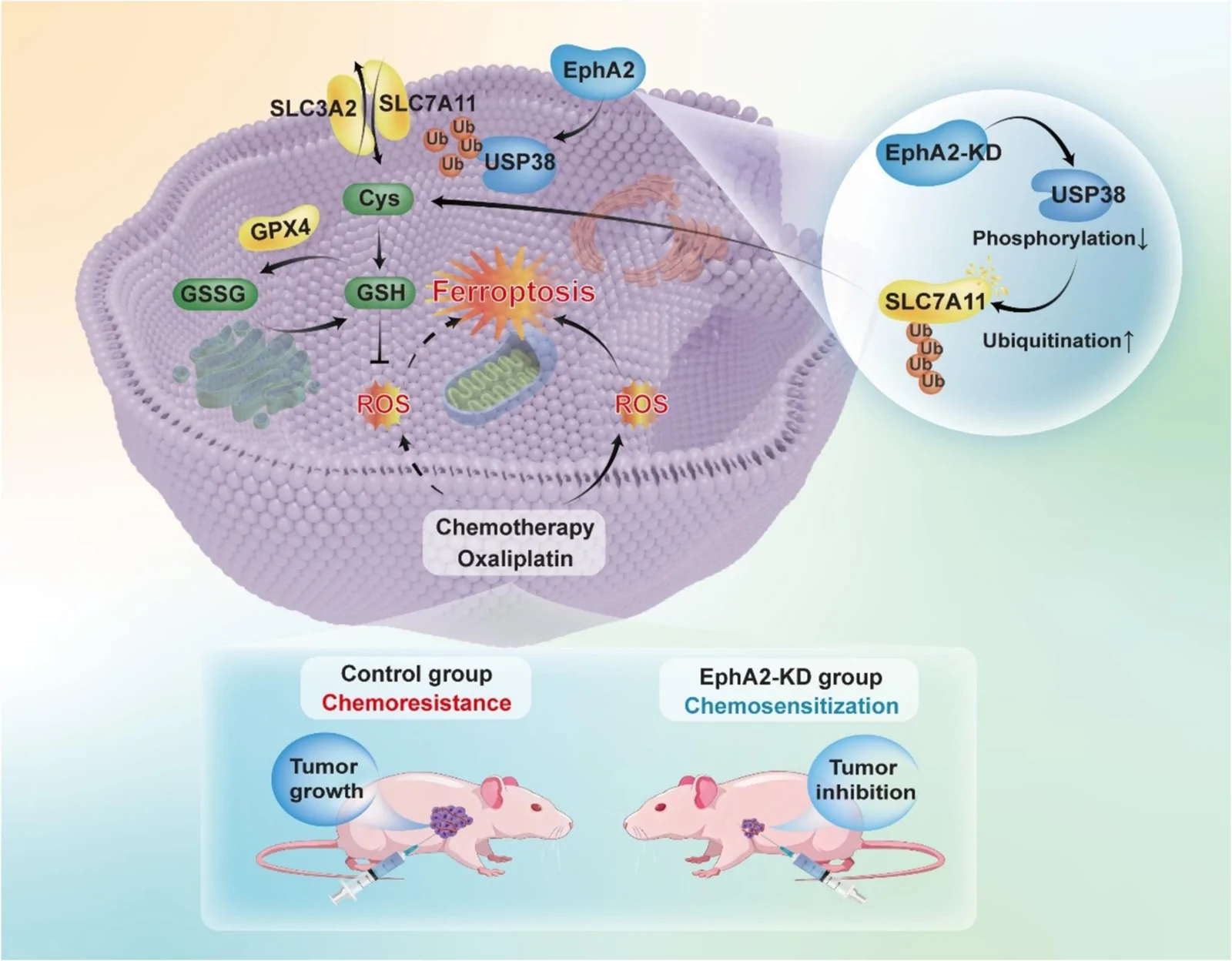
Illustration of EphA2/USP38-ROS dependent chemoresistance pattern in GC. The inhibition of EphA2 accelerated USP38-mediated degradation of SLC7A11 which could negatively regulate GC cells drug resistance to oxaliplatin through a lipid-ROS dependent pathway. Furthermore, this effect suppressed tumor growth in GC CDX-model under L-OHP treatment

A schematic model summarizing the proposed mechanism is presented in Fig. 8. Inhibition of EphA2 accelerates the USP38-dependent destabilization of SLC7A11, thereby disrupting cysteine uptake and glutathione synthesis. This leads to excessive lipid ROS accumulation, induction of ferroptosis, attenuation of chemoresistance, and suppression of tumor growth through a lipid ROS-dependent pathway. Both in vitro and in vivo experiments demonstrate that EphA2 inhibition markedly enhances ferroptosis sensitivity, exacerbates oxidative stress, and restrains GC growth, highlighting the EphA2-USP38/ROS axis as a critical determinant of chemoresistance.

## Discussion

Currently, neoadjuvant chemotherapy remains the cornerstone treatment for resectable gastric cancer (GC) [29], but chemoresistance driven by tumor heterogeneity and metabolic reprogramming significantly limits therapeutic efficacy, which leads to high mortality [30, 31]. Oxaliplatin, a third-generation platinum drug widely used in regimens like FOLFOX/XELOX, exerts anticancer effects through DNA damage and oxidative stress induction [32]. Notably, its efficacy is closely linked to the cellular redox balance. Oxaliplatin induces cytotoxicity primarily by triggering excessive reactive oxygen species (ROS) accumulation and lipid peroxidation. Nevertheless, cancer cells can acquire adaptive mechanisms to withstand this oxidative stress. Our findings demonstrate that EphA2 enhances GC cell tolerance to oxaliplatin-induced oxidative damage through ROS-dependent signaling pathways, likely mediated by metabolic reprogramming. However, the precise molecular mechanism by which EphA2 protects GC cells from ferroptosis has remained incompletely defined. Importantly, this mechanism may extend beyond oxaliplatin, suggesting a broader role for EphA2 in modulating oxidative stress-associated chemoresistance across diverse therapeutic contexts. Ferroptosis, driven by lipid peroxidation, has emerged as a therapeutic strategy against chemoresistance [33], particularly in oxaliplatin-treated cancer [34, 35]. Since excessive accumulation of lipid ROS is a hallmark of ferroptosis [36], this vulnerability is exploited by chemotherapeutic agents such as oxaliplatin and doxorubicin to induce cancer cell death [37]. In response, cancer cells may reinforce redox homeostasis by reprogramming ROS-regulatory machinery, thereby contributing to the development of chemoresistance. Of note, our study revealed a strong correlation between elevated EphA2 expression and the activation of oxidative stress-response pathways, which may partially account for the unfavorable prognosis observed in GC. Mechanistically, EphA2 appears to facilitate the activation of antioxidant defenses, thereby suppressing lethal ROS accumulation. Consistent with this notion, inhibition of EphA2 effectively abrogated this protective effect. Moreover, confocal laser scanning microscopy (CLSM) analysis demonstrated that EphA2 knockdown markedly exacerbated mitochondrial membrane damage and disrupted mitochondrial cristae integrity, further supporting a critical role for EphA2 in preserving mitochondrial homeostasis under oxidative stress conditions.

Cellular energy metabolism constitutes the central event of physiological activities, with its precise regulatory mechanism critically dependent on the maintenance of redox homeostasis. When redox equilibrium is disrupted, excessive accumulation of ROS triggers ferroptosis. EphA2, a member of the receptor tyrosine kinase family, has been implicated in a wide range of pathological processes, including tumor progression and therapy resistance [38]. As a key signal transducer, EphA2 functions as an important intracellular signaling activator by integrating extracellular cues with downstream molecular pathways [4], initiating phosphorylation modifications related to numerous key enzymes’ activation [39]. Tyrosine kinases have been widely reported to inhibit apoptosis, thereby enabling cancer cells to evade chemotherapy-induced cell death [40]. SLC7A11, the rate-limiting cystine transporter for glutathione (GSH) synthesis, functions as a central metabolic hub linking antioxidant defense to chemoresistance [41–43]. Accordingly, pharmacological or genetic inhibition of SLC7A11 has emerged as an effective strategy to restore chemosensitivity in multiple cancer types [44, 45]. In line with these observations, our results demonstrate that EphA2 enhances the pan-tyrosine phosphorylation of the deubiquitinase USP38 in GC, leading to the stabilization of SLC7A11 through suppression of its ubiquitination. This metabolic reprogramming process inhibits ferroptosis, which is often regulated by the GPX4/SLC7A11-GSH system [46, 47]. Consequently, this mechanism also exposes a therapeutic vulnerability in GC, whereby disruption of EphA2 signaling or ferroptosis defense machinery may sensitize tumors to oxidative stress-induced cell death.

Protein kinase domains, which usually contains about 250–300 amino acids, catalyze the ATP-dependent phosphorylation of serine/threonine/tyrosine residues, regulating protein function in critical processes such as cell growth, differentiation, metabolism, and death [48, 49]. Aberrant regulation of kinase activity plays a critical role in cancer and various other pathological conditions [50–53], making these domains prime therapeutic targets [54, 55]. Deubiquitinases (DUBs) are responsible for removing ubiquitin tags from target proteins, thereby regulating their stability, function, and localization [56]. Dysregulation of DUBs during tumorigenesis can promote cancer cell proliferation, invasion, and metastasis. Among the various DUB families, the ubiquitin-specific protease (USP) family represents the largest and most extensively studied subfamily. USP proteins play essential roles in cellular signal transduction and protein turnover by regulating ubiquitin-mediated protein degradation pathways [57]. Through the stabilization of key metabolic enzymes and signaling proteins, USPs exert broad regulatory effects on cancer metabolism and contribute to tumor progression. For example, USP22 stabilizes PPAR-γ to drive fatty acid synthesis [58], while USP7 enhances EZH2 stability to promote tumor progression [59]. Our findings extend this paradigm by demonstrating that EphA2 regulates the tyrosine phosphorylation of USP38, and domain-mapping analyses further identified the EphA2 protein kinase domain (amino acids 606–906) as critical for mediating this protein-protein interaction. Through this mechanism, USP38 stabilizes SLC7A11 via deubiquitination, thereby reinforcing cellular resistance to oxidative stress. Collectively, the EphA2-USP38 axis delineates a phosphorylation-deubiquitination regulatory loop that is essential for sustaining redox homeostasis and driving chemoresistance in GC.

The relationship between ferroptosis and chemoresistance remains a dynamic and developing area of research [60]. Targeting ferroptosis to overcome tumor resistance to chemotherapy therefore represents an attractive therapeutic strategy [61]. In this study, both in vitro and in vivo experiments demonstrate that inhibition of EphA2 effectively restores chemosensitivity and suppresses tumor growth. Nevertheless, it should be noted that any animal model has its own limitations, as inherent genetic and individual differences make it impossible to fully simulate the environment of tumors in the human body. In addition, although our study identifies the EphA2 protein kinase domain as critical for mediating the interaction with USP38, the precise binding interface within this domain has not yet been fully defined. Future studies should define the precise kinase domain binding sites and optimize anticancer peptides (ACPs) targeting this interaction to enhance chemotherapy efficacy. In this context, anticancer peptides (ACPs), which possess advantages in specificity, membrane permeability, and structural modifiability, represent a promising avenue for cancer treatment [62, 63]. Targeting the validated EphA2 kinase domain (amino acids 606–906) may facilitate the rational design of peptide-based inhibitors to overcome oxaliplatin resistance.

In summary, we identified a previously unrecognized EphA2-USP38-SLC7A11 axis, in which EphA2 phosphorylates and activates USP38 to stabilize SLC7A11 via deubiquitination, representing a novel therapeutic vulnerability for overcoming oxaliplatin resistance in GC. These findings highlight the critical roles of metabolic reprogramming and mitochondrial morphological alterations in ferroptosis, providing a mechanistic foundation and translational rationale for developing novel therapeutic strategies to overcome chemoresistance for GC patients.

## Supplementary Information

Below is the link to the electronic supplementary material.

Supplementary Material 1 (DOCX 2.09 MB)

## Data Availability

The data supporting this study are available from the corresponding author upon reasonable request.

## Abbreviations

BSA: Bovine serum albumin
CCK-8: Cell Counting Kit-8
CDX: Cell line-derived xenograft
CHX: Cycloheximide
CI: Confidence interval
CLSM: Confocal laser scanning microscope
Cys: Cysteine
DUB: Deubiquitinase
EphA2: Eph receptor A2
FBS: Fetal Bovine Serum
Fer-1: Ferrostatin-1
GC: Gastric cancer
GPX4: Glutathione peroxidase 4
GSH: Glutathione
GSSG: Oxidized glutathione
HE: Hematoxylin/Eosin
IC_50_: Half-maximal inhibitory concentration
IHC: Immunohistochemistry
IP: Immunoprecipitation
KEGG: Kyoto Encyclopedia of Genes and Genomes
LPO: Lipid peroxidation
OS: Overall Survival
PUFA: Poly-unsaturated fatty acids
ROS: Reactive Oxygen Species
SLC7A11: Solute carrier family 7 member 11
STAD: Stomach Adenocarcinoma
TCGA: The Cancer Genome Atlas
TEM: Transmission Electron Microscopy
UPS: Ubiquitin-proteasome system
USP38: Ubiquitin-specific protease 38
4-HNE: 4-Hydroxynonenal
