# Supplementary material for: Inhibition of EphA2 triggers ferroptosis by disrupting USP38mediated SLC7A11 stabilization in chemoresistant gastric cancer
Source: Cell Mol Life Sci. 2026 Jul 30;83(1):341. doi: 10.1007/s00018-026-06345-4 (PMC13594076; doi:10.1007/s00018-026-06345-4)
Supplement: Supplementary file 1 [file 18_2026_6345_MOESM1_ESM.docx]

**Supporting information**

**Inhibiting EphA2 destabilizes USP38/SLC7A11 to trigger ferroptosis in chemoresistant gastric cancer**

**Supporting figures and tables were listed below.**

**Fig. S1. High frequency of EphA2 expression in GC.**

**(A)** Expression of EphA2 in 375 GC samples compared to 32 healthy normal samples from TCGA database. **(B)** IHC staining results indicated high frequency of EphA2 in GC tissue. **(C-D)** Western blotting results revealed a significant high expression of EphA2 in GC cells compared to normal epithelial GES-1 cell (*P* < 0.0001). **(E)** The correlation heatmap of EphA2 expression in GC patients from TCGA. The red area represents upregulated genes, the blue area represents downregulated genes. The asterisks symbol indicates significant differences between groups: ****P* < 0.001, *****P* < 0.0001.

**Fig. S2. Oxaliplatin (L-OHP) promotes lipid-ROS generation in GC cells.**

Flow cytometry using dye BODIPY 581/591 C11 was conducted to measure lipid-ROS generation. AGS cells were pre-treated with L-OHP (2 μM) or Erastin (5 μM). The results showed that the lipid-ROS of L-OHP group and Erastin group were both significantly increased (*P* < 0.001). That indicated L-OHP’s ability to promote lipid-ROS generation in GC cells. The asterisks symbol indicates significant differences between groups: ****P* < 0.001, *****P* < 0.0001.

**Fig. S3. The time-dependent cell viability of AGS cells under oxaliplatin treatment.**

The cell viability in different AGS groups, treated with L-OHP at IC_50_ concentration, was measured via CCK-8 assay at various time points (0 h, 2 h, 6 h, 12 h, 24 h, 48 h). The 95% CI of time (hour) for drug to achieving 50% cell activity are 18.17-23.53 h (AGS Blank group), 18.51-22.73 h (AGS vector group), 10.79-13.37 h (AGS EphA2-KD group). The relative viability of cells in the EphA2-KD group was found to be significantly decreased at various L-OHP concentrations (p-values for inter-group differences were statistically significant). The asterisks symbol indicates significant differences between groups: ***P* < 0.01, ****P* < 0.001, *****P* < 0.0001, while “ns” represents no significant difference.

**Fig. S4. CLMS images of Mitochondria and Lipid-ROS staining in AGS cells.**

**(A-B)** The CLMS images indicated that oxaliplatin could significantly promote the generation of lipid-ROS at the mitochondrial area of GC cells (AGS Blank + L-OHP group), compared to none significant increase in control group (AGS Blank + PBS group) (*P* < 0.01). While the inhibition of EphA2 could significantly lead to the elevation of lipid-ROS at the mitochondrial area in AGS EphA2-KD group (*P* < 0.0001). The green fluorescence represents mitochondria area in cells stained by Mito-Tracker (#C1048, Beyotime, China). And the red fluorescence represents lipid-ROS stained by BODIPY™ 581/591 C11 (#D3861, Invitrogen™, US). The asterisks symbol indicates significant differences between groups: ***P* < 0.01, *****P* < 0.0001, while “ns” represents no significant difference. Scale bar: 20 μm.

**Fig. S5. Inhibition of EphA2 increases DNA double stranded structure damage induced by L-OHP oxidative stress.**

**(A-B)** The WB results indicated a significant increase of pH2AX (a DNA damage marker protein) levels in the EphA2-KD group (*P* < 0.05). **(C)** Typical cell images from comet experiments show that the EphA2-KD group has a longer comet tail, indicating more severe nuclear damage. In the Fer-1 group treated with antioxidants, the nuclear damage was alleviated to some extent. **(D)** The statistical results of the comet experiment showed that the EphA2-KD group had a significant increase in tail DNA content, indicating more severe damage (*P* < 0.0001). The asterisks symbol indicates significant differences between groups: * *P* < 0.05, ***P* < 0.01, *****P* < 0.0001, while “ns” represents no significant difference. Scale bar: 100 μm.

**Table. S1.**

**Hallmarks of oncogenes and suppressor in gastric cancer**

| **Genes** | **Tumorigenesis mechanism** | **Relationship with EphA2** | **Promoting gastric cancer?** | | **Ref** |
| --- | --- | --- | --- | --- | --- |
| Ras | Promoting malignant behaviors such as proliferation, migration, and invasion of gastric cancer cells. | EphA2 is a direct transcriptional target of the Ras-Raf-MAPK pathway, and ligand-stimulated EphA2 attenuates the growth factor-induced activation of Ras. | Yes | [1] | |
| Wnt/β-catenin | The Wnt/β-catenin signaling pathway regulates embryonic development, cell proliferation, differentiation, and migration. It can promote the migration and invasion ability of tumor cells by regulating the expression of cytoskeletal proteins and cell polarity. It can also maintain the characteristics of tumor stem cells, enabling them to maintain their ability for self-renewal and differentiation. | EphA2 promotes the epithelial-mesenchymal transition through Wnt/β-catenin signaling in gastric cancer. | Yes | [2] | |
| c-Myc | c-MYC is one of the most important transcription factors that are centrally implicated in multiple types of cancer cells reprogramming, proliferation, and chemoresistance. | c-MYC could bind directly to the EphA2 and Wnt1 promoter to enhance their transcription. | Yes | [2,3] | |
| YES-1 | YES1 plays a pivotal role in promoting cell proliferation, survival, and invasiveness during tumor development. Recent findings indicate that YES1 expression and activation are associated with resistance to chemotherapeutic drugs and tyrosine kinase inhibitors in human malignancies. YES1 undergoes post-translational modifications, such as lipidation and nitrosylation, which can modulate its catalytic activity, subcellular localization, and binding affinity for substrate proteins. | EphA2, YES1, and ANXA2 form a signal axis, in which YES1 activated by EphA2 phosphorylates ANXA2 at Tyr24 site, leading to ANXA2 activation and increased ANXA2 nuclear distribution in gastric cancer (GC) cells. EphA2-YES1-ANXA2 pathway drives GC invasion and metastasis, targeting this pathway would be an efficient way for the treatment of GC. | Yes | [4,5] | |
| TES | TES contains three LIM domains, which are specific interacting regions for the cell adhesion and cytoskeleton regulatory proteins. TES suppresses GC by negative regulate the assembly of actin filaments and modulates cell adhesion and motility by interacting with Lamellipodin (Lpd). | N.A. | No | [6] | |
| Runx3 | Deletion of the Runx3 locus in mice resulted in hyperplasia of the gastric epithelium. This was due to the stimulation of proliferation and suppression of apoptosis, accompanied by a reduced sensitivity to TGF-beta1. In primary human gastric cancer specimens, RUNX3 is frequently inactivated by allele loss or gene silencing due to promoter hypermethylation. The tumorigenicity of human gastric cancer cell lines in nude mice decreased as the level of RUNX3 expression increased, which indicates that RUNX3 is a bona fide tumor suppressor of gastric cancers. | N.A. | No | [7,8] | |
|  |  |  |  |  | |

**Table. S2.**

**Detailed information of cell line used in study**

| **Cell line** | **Description** | **Related Ref.** |
| --- | --- | --- |
| AGS | AGS (RRID: CVCL_0139) cell line is a cancer cell line derived from a 54 years old female Homo sapiens (Human) (NCBI Taxonomy: [9606](https://www.ncbi.nlm.nih.gov/Taxonomy/Browser/wwwtax.cgi?lvl=0&id=9606)) gastric adenocarcinoma. | [9] |
| MKN-45 | MKN-45 (RRID: CVCL_0434) cell line is a cancer cell line derived from a 62 years old female Homo sapiens (Human) (NCBI Taxonomy: [9606](https://www.ncbi.nlm.nih.gov/Taxonomy/Browser/wwwtax.cgi?lvl=0&id=9606)) gastric adenocarcinoma. | [10] |
| HGC-27 | HGC-27 (RRID: CVCL_1279) cell line originates from a poorly differentiated Homo sapiens (Human) (NCBI Taxonomy: [9606](https://www.ncbi.nlm.nih.gov/Taxonomy/Browser/wwwtax.cgi?lvl=0&id=9606)) gastric adenocarcinoma with unspecified sex and age information. | [11] |
| GES-1 | HGC-27 (RRID: CVCL_EQ22) cell line originates from a 9 month Homo sapiens (Human) (NCBI Taxonomy: [9606](https://www.ncbi.nlm.nih.gov/Taxonomy/Browser/wwwtax.cgi?lvl=0&id=9606)) normal gastric epithelial with unspecified sex information. | [12] |

**Table. S3.**

**Sequences for RNAi experiments**

| **NO.** | **Target Seq** | **vector** |
| --- | --- | --- |
| Control-seq | TTCTCCGAACGTGTCACGT | GV248 |
| EphA2-RNAi-a | CAGCCTTCGGACAGACATA | GV248 |
| EphA2-RNAi-b | TGGAAGTACGAGGTCACTTAC | GV248 |
| USP38-RNAi-a | GCCTTCAAGTACAGCCTTCTT | GV248 |
| USP38-RNAi-b | CCAGAGATTCTTACTGGTGAT | GV248 |

**Table. S4.**

**EphA2 expression and patient general information statistics**

|  | **counts** | **EphA2** | | **χ2** | ***P value*** |
| --- | --- | --- | --- | --- | --- |
|  |  | **Negative** | **Positive** |  |  |
| Age  ≤65y  ＞65y |  |  |  |  |  |
|  | 94 | 57 | 37 | 0.325 | 0.569 |
|  | 31 | 17 | 14 |  |  |
| Gender  Male  Female |  |  |  |  |  |
|  | 90 | 50 | 40 | 1.768 | 0.184 |
|  | 35 | 24 | 11 |  |  |
| Tumor size  ≤5cm  ＞5cm |  |  |  |  |  |
|  | 80 | 52 | 28 | 3.095 | 0.079 |
|  | 45 | 22 | 23 |  |  |
| Neurological invasion  No  Yes |  |  |  |  |  |
|  | 62 | 43 | 19 | 5.252 | 0.022* |
|  | 63 | 31 | 32 |  |  |
| Vascular invasion  No  Yes |  |  |  |  |  |
|  | 75 | 45 | 30 | 0.05 | 0.824 |
|  | 50 | 29 | 21 |  |  |
| Tumor differentiation  High, moderate  Low, undifferentiated |  |  |  |  |  |
|  | 32 | 23 | 9 | 2.861 | 0.091 |
|  | 93 | 51 | 42 |  |  |
| TNM stage  I-II  III-IV |  |  |  |  |  |
|  | 39 | 29 | 10 | 5.393 | 0.020* |
|  | 86 | 45 | 41 |  |  |
| Chemoresistance occurrence  No  Yes |  |  |  |  |  |
|  | 79 | 53 | 26 | 5.531 | 0.019* |
|  | 46 | 21 | 25 |  |  |
| **Total** | 125 |  |  |  |  |

**Table. S5.**

**Primer sequences for RTq-PCR**

| **Gene** | **Sequence** | |
| --- | --- | --- |
| GAPDH | forward | 5′-ACACCCACTCCTCCACCTTTG-3′ |
|  | reverse | 5′-TCCACCACCCTGTTGCTGTAG-3′ |
| SLC7A11 | forward | 5′-TTTGTTGCCCTCTCCTGCTTTG-3′ |
|  | reverse | 5′-AGTGTGCTTGCGGACATGAATC-3′ |

**Table. S6.**

**Partial list of major proteins in mass spectrometry proteomics analysis**

| **Protein FDR Confidence** | **Accession** | **Gene Name** | **Sum PEP Score** | **Coverage [%]** | **# Peptides** | **MW [kDa]** | **calc. pI** |
| --- | --- | --- | --- | --- | --- | --- | --- |
| High | A0A024R1N1 | MYH9 | 221.229 | 36 | 68 | 226.4 | 5.6 |
| High | P29317 | EPHA2 | 167.775 | 48 | 37 | 108.2 | 6.23 |
| High | A0A087WVQ6 | CLTC | 122.617 | 25 | 33 | 191.9 | 5.69 |
| High | A0A1B0GVI3 | KRT10 | 84.999 | 42 | 25 | 63.3 | 5.26 |
| High | Q7Z406 | MYH14 | 76.874 | 16 | 29 | 227.7 | 5.6 |
| High | Q8WWM7 | ATXN2L | 70.036 | 28 | 23 | 113.3 | 8.59 |
| High | P02533 | KRT14 | 49.317 | 45 | 19 | 51.5 | 5.16 |
| High | P60709 | ACTB | 48.732 | 51 | 14 | 41.7 | 5.48 |
| High | Q6IQ30 | PABPC4 | 39.652 | 25 | 13 | 72.3 | 9.35 |
| High | A0A0S2Z5H3 | CLINT1 | 33.53 | 23 | 11 | 70.3 | 6.58 |
| High | Q92804 | TAF15 | 30.387 | 18 | 8 | 61.8 | 8.02 |
| High | M0QY43 | MYH14 | 25.849 | 14 | 11 | 113.7 | 7.59 |
| High | A0A0F7KYT8 | FXR1 | 23.428 | 15 | 9 | 76.2 | 6.83 |
| High | Q9BVA1 | TUBB2B | 19.793 | 21 | 8 | 49.9 | 4.89 |
| High | A0A0S2Z4G4 | TPM3 | 17.472 | 32 | 7 | 29 | 4.78 |
| High | P50402 | EMD | 16.255 | 30 | 6 | 29 | 5.5 |
| High | Q9Y3I0 | RTCB | 14.687 | 14 | 7 | 55.2 | 7.23 |
| High | A0A0D9SF53 | DDX3X | 14.55 | 10 | 6 | 81.4 | 8.07 |
| High | A0A024R228 | HNRPK | 13.37 | 15 | 5 | 51.2 | 5.64 |
| High | P23396 | RPS3 | 12.43 | 27 | 7 | 26.7 | 9.66 |
| High | K9JA46 | EL52 | 12.271 | 9 | 6 | 84.6 | 5.02 |
| High | Q8NC56 | LEMD2 | 11.194 | 12 | 5 | 56.9 | 9 |
| High | A0A024R7L8 | UPF1 | 10.9 | 5 | 5 | 124.3 | 6.61 |
| High | P38919 | EIF4A3 | 10.654 | 13 | 5 | 46.8 | 6.73 |
| High | Q8N1C8 | HSPA9 | 9.417 | 5 | 3 | 73.8 | 6.37 |
| **High** | **Q8NB14** | **USP38** | **9.341** | **6** | **5** | **116.5** | **6.21** |
| High | A0A2R8Y5V9 | TPM4 | 9.317 | 19 | 5 | 28.6 | 4.7 |
| High | P05023 | ATP1A1 | 9.209 | 7 | 6 | 112.8 | 5.49 |
| High | Q15717 | ELAVL1 | 9.068 | 12 | 3 | 36.1 | 9.17 |

**References**

[1] Macrae M, Neve RM, Rodriguez-Viciana P, et al. A conditional feedback loop regulates Ras activity through EphA2. Cancer Cell. 2005 Aug;8(2):111-8. doi: 10.1016/j.ccr.2005.07.005. PMID: 16098464.

[2] Peng Q, Chen L, Wu W, et al. EPH receptor A2 governs a feedback loop that activates Wnt/β-catenin signaling in gastric cancer. Cell Death Dis. 2018 Nov 19;9(12):1146. doi: 10.1038/s41419-018-1164-y. PMID: 30451837; PMCID: PMC6242896.

[3] Fatma H, Maurya SK, Siddique HR. Epigenetic modifications of c-MYC: Role in cancer cell reprogramming, progression and chemoresistance. Semin Cancer Biol. 2022 Aug;83:166-176. doi: 10.1016/j.semcancer.2020.11.008. Epub 2020 Nov 18. PMID: 33220458.

[4] Kook E, Chun KS, Kim DH. Emerging Roles of YES1 in Cancer: The Putative Target in Drug Resistance. Int J Mol Sci. 2024 Jan 25;25(3):1450. doi: 10.3390/ijms25031450.

[5] Mao L, Yuan W, Cai K, et al. EphA2-YES1-ANXA2 pathway promotes gastric cancer progression and metastasis. Oncogene. 2021 May;40(20):3610-3623. doi: 10.1038/s41388-021-01786-6. Epub 2021 May 3. Erratum in: Oncogene. 2022 Feb;41(8):1228-1230. doi: 10.1038/s41388-021-02163-z.

[7] Fan XY, Hu XL, Han TM, et al. Association between RUNX3 promoter methylation and gastric cancer: a meta-analysis. BMC Gastroenterol. 2011 Aug 25;11:92. doi: 10.1186/1471-230X-11-92.

[8] Bae SC, Choi JK. Tumor suppressor activity of RUNX3. Oncogene. 2004 May 24;23(24):4336-40. doi: 10.1038/sj.onc.1207286.

[9] Rogers S, Zhang C, Anagnostidis V, et al. Cancer-associated fibroblasts influence Wnt/PCP signaling in gastric cancer cells by cytoneme-based dissemination of ROR2. Proc Natl Acad Sci U S A. 2023 Sep 26;120(39):e2217612120. doi: 10.1073/pnas.2217612120.

[10] Yu M, Ren L, Liang F, et al. Effect of epiberberine from Coptis chinensis Franch on inhibition of tumor growth in MKN-45 xenograft mice. Phytomedicine. 2020 May 17;76:153216. doi: 10.1016/j.phymed.2020.153216.

[11] Zhang J, Li Y, Chen J, et al. TOB1 modulates neutrophil phenotypes to influence gastric cancer progression and immunotherapy efficacy. Front Immunol. 2024 Mar 28;15:1369087. doi: 10.3389/fimmu.2024.1369087.

[12] Wang J, Yao Y, Zhang Q, et al. Inflammatory responses induced by Helicobacter pylori on the carcinogenesis of gastric epithelial GES‑1 cells. Int J Oncol. 2019 Jun;54(6):2200-2210. doi: 10.3892/ijo.2019.4775.
